# Health and Lifestyle Factors and Dementia Risk Among Former Professional Soccer Players

**DOI:** 10.1001/jamanetworkopen.2024.49742

**Published:** 2024-12-09

**Authors:** Emma R. Russell, Donald M. Lyall, Daniel F. Mackay, Kirstie Cronin, Katy Stewart, John A. MacLean, Jill P. Pell, William Stewart

**Affiliations:** 1 School of Psychology & Neuroscience, University of Glasgow, Glasgow, United Kingdom; 2 School of Health & Wellbeing, University of Glasgow, Glasgow, United Kingdom; 3 School of Cardiovascular & Metabolic Health, University of Glasgow, Glasgow, United Kingdom; 4 Hampden Sports Clinic, Hampden Stadium, Glasgow, United Kingdom; 5 Department of Neuropathology, Queen Elizabeth University Hospital, Glasgow, United Kingdom

## Abstract

**Importance:**

Elite-level contact sport participation is associated with increased dementia risk, which may be attributable to sport-related traumatic brain injury and repetitive head impact exposure. However, the contribution of wider, potentially modifiable dementia risk factors remains uncertain.

**Objective:**

To explore the association of potentially modifiable dementia risk factors with dementia risk among former professional soccer players.

**Design, Setting, and Participants:**

This retrospective cohort study used electronic health record linkage to national electronic datasets of general and mental health hospital inpatient and day-case admissions, prescribing information, and death certification in Scotland for male former professional soccer players born between January 1, 1900, and December 31, 1990, who were aged 30 years or older on December 31, 2020, and general population control individuals matched by sex, year of birth, and area socioeconomic status. Database interrogation was performed on November 30, 2021, and data were analyzed between January 16, 2023, and July 8, 2024.

**Exposures:**

History of smoking, depression, alcohol-related disorders, diabetes, hypertension, hearing loss, and obesity coded within electronic health records.

**Main Outcomes and Measures:**

Prevalence of dementia risk factors and their association with incident dementia diagnoses were evaluated and compared between former soccer players and matched controls.

**Results:**

The final cohort consisted of 11 984 male former professional soccer players and 35 952 matched controls. Over a median 21 years (IQR, 7-34 years) of follow-up from study entry at age 30 years or older, providing a total of 1 039 848 years of follow-up, 434 former soccer players (3.62%) and 453 matched population controls (1.26%) were identified with a dementia diagnosis (hazard ratio [HR], 3.02; 95% CI, 2.54-3.58; *P* < .001). Overall, rates of general health and lifestyle dementia risk factors were similar or lower among former soccer players compared with matched controls (eg, diabetes: 4.26% vs 6.35%). Dementia risk associated with these factors among soccer players was similar to or lower than among controls (eg, hypertension: HR, 4.62 [95% CI, 3.69-5.78] vs 6.96 [95% CI, 5.64-8.59]).

**Conclusions and Relevance:**

This cohort study found no evidence that high dementia risk among former professional soccer players was associated with potentially modifiable general health and lifestyle dementia risk factors. These data support continuation of measures directed toward reducing exposure to repetitive head impacts and traumatic brain injury in sport.

## Key Points

**Question:**

Is there an association between potentially modifiable general health and lifestyle risk factors and dementia risk among former professional soccer players compared with matched general population controls?

**Findings:**

In this cohort study of 11 984 former professional soccer players and 35 952 matched general population controls in Scotland, dementia risk was higher among soccer players despite the risk associated with multiple known modifiable dementia risk factors being lower or no different compared with that among matched population controls.

**Meaning:**

This study provides further support for measures to reduce repetitive head impact and traumatic brain injury exposure in contact sport to address dementia risk.

## Teaser

This cohort study assesses the association between potentially modifiable general health and lifestyle risk factors and risk of dementia among former professional soccer players compared with matched general population controls.

## Introduction

Participation in contact sports at an elite level has been shown to be associated with increased risk of a range of neurodegenerative diseases, including dementia.^1,2,3,4^ To date, evidence from neuropathological investigations^5,6,7^ supported by epidemiologic studies^1,2,3,4^ suggests, at least in part, that this association reflects sport-related exposure to repetitive head impacts (RHIs) and traumatic brain injury (TBI). However, TBI represents just one of many potentially modifiable general health and lifestyle risk factors for dementia,^8^ with the contribution of these wider factors to dementia risk in contact sport athletes incompletely understood thus far.

Although the association between exposure to RHIs and TBI in boxing and later-life adverse brain health has long been recognized,^9^ only in more recent years have data emerged demonstrating that this association extends beyond boxing to other contact sports. Autopsy studies have frequently reported chronic traumatic encephalopathy neuropathologic change, a pathology specific to prior exposure to RHIs and TBI, in the brains of former athletes from various contact sports.^5,6,10,11,12,13^ Notably, risk of this pathology appears to be associated with contact sport career length, interpreted as a surrogate measure of risk exposure.^6^ In parallel, epidemiologic data have demonstrated high risk of a range of neurodegenerative diseases associated with contact sports participation at an elite level.^1,2,3,4,14^ For example, several studies reported high dementia risk among former professional soccer players,^2,4,15,16,17^ with risk being highest among outfield players (ie, nongoalkeeper) and those with the longest careers,^15^ suggesting that risk reflects exposure to a sport-related factor, specifically RHIs and TBI. Nevertheless, while RHIs and TBI might be considered candidate risk factors for dementia in contact sport athletes, there remain many other potential dementia risk factors that have yet to be explored in these populations.

The Lancet Commission on Dementia, Intervention, and Care identified 12 potentially modifiable general health and lifestyle risk factors for dementia,^8^ among which is history of TBI. Together, these 12 risk factors are suggested to be responsible for up to 40% of dementia in the community, raising the prospect that targeted intervention directed toward addressing these risk factors might produce a considerable reduction in the burden of disease.^18,19^ Regarding former contact sport athletes, there remain relatively few and conflicting data on these wider risk factors, with no data on their contribution to dementia risk. For example, among former professional soccer players, mortality from cardiovascular disease^2,17,20^ and lung cancer^2^ is reported as low, suggesting better cardiometabolic health and lower rates of smoking, both potentially favoring lower dementia risk. In contrast, former professional American football players are reported to have higher hypertension and diabetes rates than general population estimates^21^ and higher cardiovascular mortality than non–contact sport athletes.^22^ There is, therefore, a need to better understand the contribution of these wider dementia risk factors to dementia among at-risk contact sport athlete populations to inform appropriate risk-mitigation strategies.

Soccer is the most popular participation sport worldwide, with over one-quarter of a billion active participants across more than 200 countries.^23^ Studies of former professional soccer players reported high dementia risk in comparison with general population data^2,4,15,16,17^ and provided limited insights suggesting that former soccer players may have better outcomes in measures of general health.^2^ In this context, we hypothesized that rates of many general health and lifestyle risk factors for dementia would be lower among former soccer players than among matched general population controls and, furthermore, that the contribution of these wider risk factors to risk of dementia among former soccer players would be less than among matched controls. To address these hypotheses, we accessed national electronic health records to explore available data on rates of various dementia risk factors and their association with dementia risk among male former professional soccer players in Scotland.

## Methods

### Approval

Ethical approval for this cohort study was granted by the University of Glasgow College of Medical, Veterinary & Life Sciences Ethics Committee, with protocol and data governance procedures approved by National Health Service (NHS) Scotland’s Public Benefit and Privacy Panel for Health and Social Care. As all health record data were anonymized to researchers, individual participant informed consent was not required. The analysis and reporting of this study were consistent with the Strengthening the Reporting of Observational Studies in Epidemiology (STROBE) reporting guideline.^24^

### Cohort Identification and Inclusion Criteria

Former professional soccer players were identified through the Record of Pre-War Scottish League Players, version 2^25^ and the Record of Post-War Scottish League Players, version 6,^26^ compiled from archives of the Scottish Football Museum and individual league clubs. Study inclusion was restricted to individuals born between January 1, 1900, and December 31, 1990, and aged 30 years or older on December 31, 2020. All individuals included in this study were male, as identified by electronic health records classification. Probabilistic matching was applied to the full name and date of birth for the former professional soccer player cohort to identify their unique Community Health Index (CHI) number, with postcode-level area socioeconomic status available for all CHI matched individuals. The CHI database was also used to randomly identify population controls with male sex and match them to the former soccer players in a 3:1 ratio by year of birth and quintile of postcode-level area socioeconomic status. The NHS Information Services Division records the last known postcode of residence for all individuals, from which area socioeconomic deprivation is calculated using the Scottish Index of Multiple Deprivation (SIMD), derived from information on income, employment, health, educational level, housing, and crime.^27^ The SIMD is categorized into quintiles ranging from 1 (most deprived) to 5 (most affluent).

### Incident Dementia Diagnosis and Associated Risk Factors

Outcomes for former soccer players and the matched population controls, whether living or deceased at the time of data capture, were obtained by individual-level record linkage to hospitalizations ascertained from the Scottish Morbidity Record (SMR) 01 (General/Acute Inpatient and Day Case) and SMR04 (Mental Health Inpatient and Day Case), dispensed prescriptions ascertained from the Scottish National Prescribing Information System, and death certificates. SMR01, SMR04, and death certification datasets were available from 1981 onward and were assessed for outcomes by *International Classification of Diseases, Ninth Revision (ICD-9)* and *International Statistical Classification of Diseases and Related Health Problems, Tenth Revision (ICD-10)* coding. Dispensed prescriptions were available from the prescription information system from 2009 onward and coded in accordance with the British National Formulary. A complete list of the *ICD-9*, *ICD-10*, and British National Formulary Section codes used to ascertain incident dementia diagnoses and potentially modifiable dementia risk factors is provided in Table 1. For the purposes of this study, datasets were searched for dementia diagnoses coded as vascular dementia; dementia in other diseases classified elsewhere; unspecified dementia; degenerative diseases of basal ganglia; Alzheimer disease; other degenerative diseases of the nervous system, including but not limited to frontotemporal dementia, senile degeneration, and dementia with Lewy bodies; and dementia not otherwise specified. Regarding the potentially modifiable dementia risk factors identified by the 2020 Lancet Commission,^8^ information relevant to smoking, depression, diabetes, alcohol-related disorders, hypertension, hearing loss, and obesity was available via electronic health records. All outcomes were ascertained from the earliest coding of a disease or disorder from related hospital attendance, prescribing information, or death certification.

**Table 1.** Disease and Medication Codes

Disease	Datasets	*ICD-9* codes	*ICD-10* codes	BNF code
Dementia	Death certificates, SMR01, SMR04, PIS	290.0-290.4, 294.1, 294.2, 331.0, 331.1, 331.2, 331.6-331.9	F01-F03, G23.1-G23.3, G30, G31	4.11
Hypertension	SMR01	401-405	I10-I16	NA
Diabetes	SMR01, PIS	250	E08-E13	6.1
ARD	SMR01, SMR04	303	F10	4.10.1
Smoking	SMR01, SMR04	305.1	F17	4.10.2
Depression	SMR01, SMR04	296.2-296.3	F32-F33	NA
Hearing loss	SMR01	389	H90-H94	NA
Obesity	SMR01, PIS	278	E66	4.5

Abbreviations: ARD, alcohol-related disorder; BNF, British National Formulary; *ICD-9*, *International Classification of Diseases, Ninth Revision*; *ICD-10*, *International Statistical Classification of Diseases and Related Health Problems, Tenth Revision*; NA, not applicable; PIS, Prescribing Information System; SMR, Scottish Morbidity Record.

### Statistical Analysis

All analyses included data up to December 31, 2020, with database interrogation performed on November 30, 2021. Cox proportional hazards regression analysis was used to assess difference in risk of dementia between former professional soccer players and matched population controls, with assumptions tested using Schoënfeld residuals. Thereafter, risk of dementia associated with each identified potentially modifiable risk factor was assessed univariately in former soccer players and matched controls. Results are reported as hazard ratios (HRs) and 95% CIs. The association between individual risk factors and dementia outcomes among former soccer players and matched general population controls were compared by performing a 2-way interaction analysis using a logistic regression model to assess for any interaction. In addition, for each subgroup, population-attributable fractions associated with each potentially modifiable risk factor were estimated using the punafcc command in Stata, version 16 (StataCorp LLC).^28^ All statistical analyses were undertaken between January 16, 2023, and July 8, 2024, using Stata, version 16,^28^ with statistical significance set at 2-sided *P* < .05.

## Results

### Dementia and Other Health Outcomes Among Former Professional Soccer Players

In total, the names and dates of birth of 16 473 former soccer players were submitted to Public Health Scotland for CHI matching. Of these, 1195 were excluded as duplicate entries, and the CHI matching process was unsuccessful for a further 3294 due to incomplete or inaccurate demographic data. As a result, the final cohort included in health records interrogation consisted of 11 984 former professional soccer players and 35 952 general population controls individually matched by male sex, exact area socioeconomic status, and year of birth. Over a median of 21 years (IQR, 7-34 years) of follow-up from study entry at the age of 30 years or older, providing a total of 1 039 848 years follow-up, 434 of the former soccer players (3.62%) and 453 of the matched general population controls (1.26%) were identified with an incident dementia diagnosis (HR, 3.02; 95% CI, 2.54-3.58; *P* < .001). Regarding potentially modifiable risk factors for dementia, overall rates of these factors were similar or lower among former soccer players compared with matched controls (eg, diabetes: 4.26% vs 6.35%). Reflecting this, former soccer players had lower risk of alcohol-related disorders (HR, 0.77; 95% CI, 0.69-0.87; *P* < .001), smoking (HR, 0.55; 95% CI, 0.46-0.66; *P* < .001), diabetes (HR, 0.75; 95% CI, 0.67-0.85; *P* < .001), and obesity (HR, 0.72; 95% CI, 0.59-0.87; *P* = .001) and similar risk of hearing loss, depression, and hypertension compared with matched general population controls (Table 2).

**Table 2.** Comparison of Rates of Dementia and Potentially Modifiable Risk Factors Between Former Soccer Players and Matched Controls

Risk factor	Participants, No. (%)		HR (95% CI)	*P* value^a^
	Former soccer players (n = 11 984)	Matched controls (n = 35 952)		
Dementia	434 (3.62)	453 (1.26)	3.02 (2.54-3.58)	<.001
Hypertension	884 (7.38)	2678 (7.45)	0.97 (0.88-1.06)	.51
Diabetes	511 (4.26)	2283 (6.35)	0.75 (0.67-0.85)	<.001
ARD	482 (4.02)	1778 (4.95)	0.77 (0.69-0.87)	<.001
Smoking	170 (1.42)	910 (2.53)	0.55 (0.46-0.66)	<.001
Depression	138 (1.15)	513 (1.43)	0.85 (0.69-1.04)	.12
Hearing loss	131 (1.09)	369 (1.03)	1.05 (0.85-1.28)	.66
Obesity	128 (1.07)	533 (1.48)	0.72 (0.59-0.87)	.001

Abbreviations: ARD, alcohol-related disorder; HR, hazard ratio.

^a^ From Cox proportional hazards regression.

### Potentially Modifiable Risk Factors and Risk of Dementia

In the general population control group, increased risk of dementia was observed in association with history of hypertension, diabetes, alcohol-related disorders, smoking, depression, and hearing loss, with risk greatest for the association with hypertension (HR, 6.96; 95% CI, 5.64-8.59; *P* < .001). In contrast, among former soccer players, increased dementia risk was only observed in association with hypertension (HR, 4.62; 95% CI, 3.69-5.78; *P* < .001) and depression (HR, 1.93; 95% CI, 1.23-3.02; *P* = .004), with the remaining potentially modifiable risk factors showing no association with dementia risk (Table 3). Regarding interactions of dementia risk with potentially modifiable risk factors, the effect sizes were smaller for hypertension (*P* = .04 for interaction), alcohol-related disorders (*P* < .001 for interaction), and smoking (*P* = .02 for interaction) among former soccer players compared with the control group. Consistent with this, the population-attributable fraction for dementia associated with hypertension was higher among population controls (13.10%; 95% CI, 7.40%-18.43%) than among former soccer players (3.79%; 95% CI, −1.87% to 9.13%); similar findings were observed in population-attributable fractions for alcohol-related disorders and diabetes (Table 4).

**Table 3.** Potentially Modifiable Risk Factors and Associated Risk of Dementia Among Former Soccer Players and Controls

Risk factor	Former soccer players		Matched controls	
	HR (95% CI)	*P* value^a^	HR (95% CI)	*P* value^a^
Hypertension	4.62 (3.69-5.78)	<.001	6.96 (5.64-8.59)	<.001
Diabetes	1.04 (0.77-1.41)	.78	1.38 (1.09-1.74)	.007
ARD	1.22 (0.84-1.77)	.29	2.92 (2.19-3.88)	<.001
Smoking	0.67 (0.30-1.51)	.34	1.84 (1.19-2.86)	.006
Depression	1.93 (1.23-3.02)	.004	3.43 (2.27-5.18)	<.001
Hearing loss	1.72 (0.89-3.34)	.11	1.98 (1.06-3.70)	.03
Obesity	0.57 (0.18-1.77)	.33	1.26 (0.67-2.37)	.47

Abbreviations: ARD, alcohol-related disorder; HR, hazard ratio.

^a^ From Cox proportional hazards regression.

**Table 4.** Population-Attributable Fraction of Dementia Associated With Potentially Modifiable Risk Factors

Risk factor	Population-attributable fraction, % (95% CI)	
	Former soccer players	Matched controls
Hypertension	3.79 (−1.87 to 9.13)	13.10 (7.40 to 18.43)
Diabetes	0.90 (−2.37 to 4.16)	7.13 (2.86 to 11.21)
ARD	0.60 (−1.90 to 3.08)	6.00 (2.90 to 9.16)
Smoking	−0.76 (−1.93 to 0.39)	1.98 (−0.01 to 3.93)
Depression	2.55 (0.66 to 4.41)	3.59 (1.51 to 5.63)
Hearing loss	1.26 (0.07 to 2.43)	1.13 (−0.24 to 2.49)
Obesity	−0.77 (−1.68 to 0.14)	0.60 (−1.34 to 1.44)

Abbreviation: ARD, alcohol-related disorder.

## Discussion

While there have been several studies demonstrating increased risk of neurodegenerative disease in former contact sport athletes,^1,2,3,4^ to date, these reports have largely failed to consider the role of potentially modifiable general health and lifestyle risk factors in contributing to the neurodegenerative disease risk observed. In this retrospective cohort study comparing electronic health record data on outcomes in a large cohort of male former professional soccer players in Scotland with those of a matched general population comparison group, we observed that while dementia risk was higher among former soccer players, rates of many potentially modifiable dementia risk factors were comparable, if not lower. Compared with matched general population controls, rates of diabetes, obesity, smoking, and alcohol-related disorders were lower among soccer players, while rates of hypertension, hearing loss, and depression were similar in both groups. Furthermore, the contribution of these general health and lifestyle factors to the risk of dementia among soccer players was less than among matched population controls. As such, while revealing high risk of dementia among soccer players, these data also suggest that this association was not attributable to wider general health and lifestyle factors recognized as dementia risk factors.

Increased risk of neurodegenerative disease associated with elite-level contact sport participation has consistently been reported across multiple studies of former athletes from various contact sports, including soccer,^2,4,15,17^ rugby union,^3,6^ and American football.^1,29^ Regarding soccer, risk of dementia among soccer players in the current analysis is in line with our group’s previous analyses reporting neurodegenerative disease mortality^2^ and incident neurodegenerative disease diagnoses^15^ among Scottish former professional soccer players, with those previous studies reporting data for a smaller cohort captured with a higher age at inclusion and with data censored at December 31, 2016. In our current analysis, we included former soccer players aged 30 years or older at study inclusion, with data censored at December 31, 2020. The present, larger study population, therefore, included participants from our group’s earlier reports.^2,15^ Notably, dementia risk observed in the current and previous retrospective cohort studies of Scottish former soccer players^2,15^ is in line with data on standardized mortality rates among French former professional soccer players^17^ and self-reporting of dementia in a cross-sectional study of English former professional soccer players.^16^ As such, there are now several studies using varying methods and in geographically distinct populations corroborating a 3- to 3.5-fold increased dementia risk among former professional soccer players compared with general population data.

Regarding the factors associated with the observed dementia risk among former contact sport athletes, our group previously found that risk among former soccer players was associated with player position and length of playing career, with outfield positions and longest playing careers being associated with highest dementia risk.^15^ Elsewhere, higher self-reported estimates of heading frequency have been reported to be associated with increased risk of cognitive impairment among former soccer players.^30^ Furthermore, multiple autopsy studies of former contact sport athletes, including former soccer players, reported the frequent finding of a specific neurodegenerative pathology, chronic traumatic encephalopathy neuropathologic change, intimately associated with prior exposure to TBI and RHIs.^5,6,7,11,12,13^ Taken together, these parallel observations are interpreted as evidence that a sport-associated factor (specifically, exposure to TBI or RHIs) may contribute to dementia risk. However, multiple other factors are known to be associated with dementia risk, including potentially modifiable lifestyle and general health risk factors for dementia.^8,18^

The 2020 Lancet Commission on Dementia Prevention, Intervention, and Care cited 12 potentially modifiable risk factors for dementia, including TBI.^8^ NHS Scotland’s electronic health records provide data suitable for insight into 7 of these 12 risk factors. Compared with matched general population controls, the former professional soccer players in this study had similar if not lower rates of these risk factors. In other words, in wider health and lifestyle measures, the former soccer players generally were healthier with respect to potentially modifiable dementia risk factors. Furthermore, the contribution of these risk factors to observed dementia rates among soccer players in this study was less than among matched controls. These findings are broadly in line with research demonstrating lifelong general health benefits of high levels of physical activity associated with participation in sports at an elite level.^31,32^ Nevertheless, despite the demonstrable general health and lifestyle benefits observed among the former professional soccer players in this study, which conceivably might be expected to be associated with lower dementia risk, our data showed high dementia risk in this population.

Because of the observed association between contact sports and lifelong brain health outcomes, specifically neurodegenerative disease risk, there are several initiatives across sports to act to mitigate risk when practical. As such, with evidence to date suggesting that this disease risk is associated with exposure to a sport-associated factor, specifically TBI or RHIs, there are ongoing efforts to reduce unnecessary exposure to RHIs and TBI and better recognize and manage sport-associated TBI when it occurs.^33,34,35^ In addition to these primary prevention measures, there are various initiatives intended as secondary prevention to mitigate dementia risk in former athletes. Initiatives, such as Brain Health Scotland, have been set up in an attempt to detect early signs of neurodegenerative disease and work alongside individuals of all ages to develop their own dementia prevention plans through lifestyle alterations.^36^ One challenge presented by the current data might be that in the context of generally better dementia risk profiles with respect to wider health and lifestyle factors, advice on risk mitigation based on data gathered in research and clinical practice in general, nonathlete populations may have less relevance to these former contact sport athletes. Future research should seek to identify tests that detect earliest indices of evolving neurodegeneration prior to clinical disease presentation in contact sport athletes, allowing for timely interventions to reduce dementia risk or delay onset of the disease.^37^ In the interim, continued interventions directed toward risk mitigation addressing recognized, potentially modifiable risk factors should continue.

### Strengths and Limitations

A strength of this work is inclusion of a large, comprehensive cohort comprising 11 984 male former professional soccer players, each matched with 3 population controls on age, sex, and socioeconomic status. By matching for area socioeconomic status, we controlled for potential biases attributable to access to the NHS and limited private health care in Scotland. Furthermore, our analyses comparing dementia risk associated with various wider health and lifestyle factors among former professional soccer players to some extent addresses the potential confounder of healthy worker bias.

This study also has limitations. Our analyses lacked certain variables that are purported risk factors for dementia. Pollution data for this cohort were unavailable. Pollution (typically indexed by fine particulate matter of 2.5) is fundamentally complex to analyze; a single cross-sectional snapshot (eg, home postcode) is unlikely to give an accurate estimate of an individual’s average lifetime exposure. Consideration of multiple key confounders (eg, time spent outdoors, proximity to roads, or pollution at the workplace) is important.^38^ Thus, in this context, pollution was not a practical exposure to consider. We did not consider the potential association of educational attainment in this cohort. While lower educational attainment is a recognized potential factor associated with dementia,^8^ inclusion of educational attainment as a covariate in exposure and outcome associations often makes relatively little difference to estimates.^39^ It is also important to note that this study did not include information on individuals managed exclusively in a primary care setting. In an ideal study setting, information regarding health outcomes managed in a primary care setting would also be available. Nevertheless, we have no reason to expect systematic bias to one group or another in the level of health care accessed for the outcomes assessed.

## Conclusions

In this cohort study, compared with matched general population controls, our data showed former professional soccer players had similar or lower risk of multiple common, potentially modifiable risk factors for dementia. Specifically, rates of hypertension, diabetes, depression, hearing loss, obesity, smoking, and alcohol-related disorders were lower among former soccer players than among their matched controls. The findings suggest that while interventions to address general health and lifestyle risk factors remain recommended, the priority for neurodegenerative disease risk mitigation among contact sport athletes should continue to be reduction, if not removal, of exposure to RHIs and TBI when practical.

## Notes

Supplement.
Data Sharing Statement
