# Supplementary material for: Health and Lifestyle Factors and Dementia Risk Among Former Professional Soccer Players
Source: JAMA Netw Open. 2024 Dec 9;7(12):e2449742. doi: 10.1001/jamanetworkopen.2024.49742 (PMC11629125; doi:10.1001/jamanetworkopen.2024.49742)
Supplement: Supplement. — Data Sharing Statement [file jamanetwopen-e2449742-s001.pdf]

Data Sharing Statement

Russell. Health and Lifestyle Factors and Dementia Risk Among Former Professional Soccer Players. *JAMA Netw Open*. Published December 09, 2024.
doi:10.1001/jamanetworkopen.2024.49742

Data
Data available: No

Additional Information
Explanation for why data not available: Data available through NHS Research Scotland Public Benefit and Privacy Panel application
